# Phytochemical compounds, antioxidant activity, and antiproliferative activity of sesame seeds as affected by simulated digestion^☆^

**DOI:** 10.1016/j.fochx.2025.103317

**Published:** 2025-11-26

**Authors:** Lin Zhou, Xiaohui Lin, Ruixue Guo, Tong Li, Charles Brennan, Xiong Fu, Rui Hai Liu

**Affiliations:** a School of Life Science and Biopharmaceutics, Guangdong Pharmaceutical University, Guangzhou 510006, PR China; b School of Food Science and Engineering, South China University of Technology, Guangzhou 510640, PR China; c School of Biosystems and Food Engineering, University College Dublin (UCD), Belfield, Dublin 4, Ireland; d Ghent University, Center for Microbial Ecology and Technology, B-9000 Gent, Belgium; e Department of Food Science, Cornell University, Ithaca, NY 14850-2824, USA; f School of Science, RMIT University, Melbourne, VIC 3000, Australia

**Keywords:** Sesame seeds, Simulated digestion, Antioxidant activity, Cell proliferation

## Abstract

Antioxidant and antiproliferative activities in white and black sesame seeds were investigated during a simulated *in vitro* digestion. The levels of phenolic compounds, flavonoids and oxygen radical absorbance capacity (ORAC) values of sesame seeds increased by over 50 % after simulated stomach, small and large intestine digestion. A higher cellular antioxidant activity (CAA) and a higher inhibition of HepG2 cell proliferation were found in the extract from small intestine digestion phase. In comparison with Aijiao Bawangbian (white color), the phenolics, flavonoids, ORAC values, CAA values and antiproliferative activity of Changzhi II (black color) were higher both before and after simulated digestion. In tested phenolics, sesamol and ferulic acid showed better antioxidant and antiproliferative activities than pinoresinol diglucoside, pinoresinol, sesamolin, and sesamin in cellular level. Sesame seed has considerable cellular antioxidant and antiproliferative activities both before and after simulated digestion, which merits further investigation *in vivo* studies.

## Highlights

• *In vitro* gastrointestinal digestion increased bioactivity of sesame seeds.
• Intestine digestion increased the release of phenolics and flavonoids.
• Sesamol and ferulic acid showed better antioxidant and antiproliferative activities.

## 1 Introduction

Sesame seeds contain around 50 % oil which is a good source of polyunsaturated fatty acids (Xiang et al., 2025). Vitamin E, sesamin, sesamolin, black pigment and other bioactive compounds in sesame seeds showed different biological activities (Jaffar et al., 2025). The natural antioxidants including sesamol, sesamin, and sesamolin in sesame seed could reduce lipid oxidation and free radicals (Suja et al., 2004). Sesaminol glucosides could protect cells from Aβ-induced neuronal cell death *via* antioxidative capacity (Lee et al., 2005). Ferulic acid were found to have several physiological functions, such as antioxidant, antithrombosis, and anticancer activities (Ou & Kwok, 2004). Pinoresinol has displayed significant antioxidant activity (Leu et al., 2011). Additionally, pinoresinol, pinoresinol monoglucoside and pinoresinol diglucoside were precursors of enterolactone which had significant preventative effects against various cancers, including breast, prostate, cervical, and colon cancers (Yan et al., 2015).

A study reported that sesame showed preventive effect on hyperglycemia and obesity in rats (Bigoniya et al., 2012). However, another study has suggested that sesame supplementation failed to improve cardiovascular disease risk markers in overweight people (Wu et al., 2009). Since sesame seeds were reported to display different bioactivities through *in vivo* studies, it is important to investigate the influence of digestion on sesame seeds. The antioxidant properties of foods have been reported widely, however, the gastrointestinal digestion effect on the food bioactivity still needs further investigation. Only antioxidants reserved after digestion might be bioavailable for uptake into human body and exert their biological function (Pinacho et al., 2015). The digestion processes modifies the physical structure of food regulating the bio-accessibility as well as the antioxidant capacity of bound phytochemicals in the gastrointestinal environment (Papillo et al., 2014). Different foods showed different transformation after gastrointestinal digestion. Thus, it is important to study the difference of specific food products before and after *in vitro* digestion. Most studies have investigated the bioactive compounds and chemical antioxidant activities of different sesame seeds after *in vitro* gastrointestinal digestion. However, the cellular antioxidant and anti proliferation activity of sesame seeds after *in vitro* gastrointestinal digestion are lack of investigation (Li et al., 2024). In our previous study, both white and black sesame seeds present considerable antioxidant and antiproliferative activities before gastrointestinal digestion (Lin et al., 2017; Zhou et al., 2016). Black sesame varieties exhibited higher total phenolic content and stronger antioxidant and antiproliferative activity compared to white sesame seeds. It is important to know whether black sesame seeds retain higher bioactive activities after digestion. Therefore, black and white sesame seeds were selected to compare their phenolic compounds, flavonoids, antioxidants and antiproliferative activities before and after *in vitro* simulated digestion. The results of the study will be helpful for the utilization and popularization of the functional components of black and white sesame seeds and provide the basis for the evaluation of sesame germplasm resources.

## 2 Material and methods

### 2.1 Plant materials

Changzhi II (black sesame seed) was purchased from Jiangxi Huanong seed company (Jiangxi, China). Aijiao Bawangbian (white sesame seed) was purchased from Henan Aoruite Seed Technology Co. Ltd. (Henan, China).

### 2.2 Free phytochemical compound extraction

Sesame seeds were ground into powder, and free phytochemical compounds were extracted (Chu & Liu, 2005). Sesame seeds (3 g) were blended in *n*-hexane and was centrifuged at 5000 rpm for 3 min. The paste was blended in 80 % acetone and was centrifuged. The solvent was collected, while the residue was blended and centrifuged twice again. The collected supernatants were concentrated to 3 mL and then 7 mL water was added.

### 2.3 Acquisition of bound phytochemical compounds

Bound phytochemical compounds were collected as explained in our previous study (Lin et al., 2017). Residues from Section 2.2 were mixed with 10 mL sodium of 4 mol/L hydroxide and were shaken for 1 h. The pH was adjusted to 7. The residue was then extracted three times by ethyl acetate. The acquired supernatant was concentrated to 3 mL and water was added to reach 10 mL.

### 2.4 *In vitro* digestion of sesame seeds

Sesame seeds were digested using a sequential multistep enzymatic treatment described by Papillo et al. (2014). This method offers a controlled framework to assess relative stability and interactions of particles under physiologically relevant conditions. The chosen parameters (pH adjustments, enzyme concentrations, and incubation times) represent average physiological ranges but may differ from *in vivo* conditions in terms of temporal dynamics and compositional variability. Therefore, the results should be interpreted as indicative rather than predictive of *in vivo* behavior.

Two grams of sesame seeds were added to test tube 1–4, respectively. Alpha-amylase (65 mg) were added in 50 mL of 2 mM CaCl_2_ solution. The α-amylase solution (500 μL) and 20 mL water were added to the sample tubes 1–4. After adjusting the pH to 2 (tubes 2–4) with HCl, pepsin (0.1 g) was mixed with test tubes 2–4, which were incubated for 60 min (gastric phase). The pH of the mixture in tubes 3–4 was adjusted to 7.4. Small intestines solution was prepared with 0.l g pancreatin, 0.25 g lipase, 1.25 g bile salts and 50 mL of 0.l mol/L NaHCO_3_ solution and was added to tubes 3 and 4. The mixture was incubated for 120 min at 37 °C. Tube 4 was modified to pH = 4 and was mixed with 50 μL Viscozyme L. The tube was incubated for 14 h at 37 °C (large intestine). All tubes were centrifuged to collect the supernatants.

### 2.5 Total phenolic content detection

Total phenolics were detected using Folin-Ciocalteu colorimetric. In brief, Folin-Ciocalteu reagents (0.1 mL) and 400 μL water were mixed with 0.1 mL sample or gallic acid (GAE). After standing for 6 min, the tubes were added with 1 mL NaCO_3_ solution (7 %) and 800 μL deionized water. After 90 min, the solution was measured at 760 nm. The unit of mg GAE/100 g DW (dry weight) was used to show calculation results.

### 2.6 Total flavonoid content detection

The total flavonoid content was analyzed by sodium borohydride-chloranil reported previously (Wang et al., 2016). Tetrahydrofuran (500 μL) and ethanol (500 μL) were added to samples and catechin (CE) standards, respectively. Then, they were added with 500 μL of 50 mM NaBH_4_ solution and 500 μL of 74.6 mM AlCl_3_ solution. The mixture was shaken for 0.5 h. NaBH_4_ was mixed with the solution and was shaken for 0.5 h. Cold acetic acid (0.8 mM, 2 mL) was added to solution. The mixture was shaken in the dark for 15 min, one mL of 20.0 mM chloranil was added to the solution and kept at 95 °C for 60 min. The mixture was mixed with 2 mL of 12 mL HCl and was centrifuged at 3500 rpm and 3 min. The upper layer was collected, and the absorbance was acquired at 490 nm. The unit of the reported data was mg CE/100 g DW.

### 2.7 Analysis of six phenolic compounds

Analysis of sesamol, pinoresinol diglucoside, pinoresinol, ferulic acid, sesamolin, and sesamin were detected by a Waters HPLC system (Waters Corp., Milford, MA, USA) equipped with a binary pump (model 1525), a C18 reversed phase column (4.6 × 250 mm, 5 μm of particle size, Waters, USA), an auto sampler (model 2707), and a Photodiode Array detector (PAD) (Waters 2998). The solution (30 μL) included solvent T (0.1 % trifluoroacetic acid, *v*/v) and solvent M (methanol), and a gradient elution program was used: 0–10 min 5 % M, 10–26 min 5–30 % M, 26–29 min 30–40 % M, 29–49 min 40–60 % M, 49–50 min 60–75 % M, 50–65 min with 75 % M, 65–67 min with 75–5 % M. The flow rate of 0.8 mL/min was used. The absorption at 270, 285, 290 and 323 nm were monitored.

### 2.8 Total antioxidant activities

ORAC assay was applied to evaluate antioxidant activities (Lin et al., 2017). Extracts or 6-hydroxy-2,5,7,8-tetramethylchronman-2-carboxylic acid (TE) were added with 20 μL of μM Fluorescein in a 96-well plate. The plate was put at 37 °C for 20 min and was added with 20 μL of 119.4 mM 2,2′-azobis-amidinopropane (ABAP). Fluorescence measurements were recorded at an excitation wavelength of 485 nm and an emission wavelength of 535 nm every 5 min for 2.5 h at 37 °C.

### 2.9 Cellular antioxidant activity analysis

HepG2 cells were seeded on a 96-well for 24 h and were washed with 100 uL PBS. Samples (100 μL) or quercetin (QE) containing 50 μM dichlorofluorescin diacetate were added to the plate and kept in 37 °C 1 h. Two protocols named PBS washing and no PBS washing were conducted with and without PBS washing before adding ABAP (100 μL, 600 μmol/L in HBSS). The absorbance was evaluated at 485 nm excitation and 535 nm emission.

### 2.10 Cytotoxicity and antiproliferative activity

The cytotoxic effects were assessed using a methylene blue assay (Zhang et al., 2022). HepG2 was cultured in the plate with 4 × 10^4^ cells per well and incubated at 37 °C for 1 day. The wells were added with samples and incubated for another 24 h. The cells were incubated with 50 uL methylene blue solution (1.25 % glutaraldehyde, and 0.6 % methylene blue in HBSS) at 37 °C for 30 min. After the plate was washed by ultra pure water, elution buffer (0.1 mL, 1 % acetic acid and 50 % ethanol in PBS) was added to mixtures. The plate was evaluated at 570 nm using plate reader. The cytotoxicity was interpreted as cytotoxicity concentration 50 % (CC_50_).

The antiproliferative activities of extracts were determined using methylene blue method (Felice et al., 2009). Samples were added to the HepG2 after 5 h incubation. After 72 h, cells were incubated with methylene blue for 60 min and washed by water. Elution buffer was added to wells. The absorbance value was detected at 570 nm. Antiproliferative activity was displayed as effective dose 50 % (EC_50_).

## 3 Results and discussion

### 3.1 Total phenolics and flavonoids

Table 1 presents the impact of simulated gastrointestinal digestion on the total flavonoid and phenolic levels of sesame seeds. After digestion, the phenolic content of Changzhi II and Aijiao Bawangbian was 113.54–790.34 mg GAE/100 g DW, and 105.14–530.46 mg GAE/100 g DW, respectively. The phenolic content was lowest in the oral phase and highest in the small intestine phase. The phenolics released in gastric, small intestine and large intestine phases were significantly higher than the total phenolics extracted by the chemical solvent. The phenolic contents in Changzhi II were higher than that of Aijiao Bawangbian before and after gastrointestinal digestion. After the digestion, the flavonoid contents of Changzhi II and Aijiao Bawangbian ranged from 162.52 (oral phase) to 1827.17 (small intestine phase) mg CE/100 g DW and from 74.40 (oral phase) to 834.15 (large intestine phase) mg CE/100 g DW, respectively. Flavonoids of Changzhi II released at the small intestine phase were significantly higher than the total flavonoids extracted by the chemical solvent. The flavonoids of Aijiao Bawangbian released in gastric, small and large intestine phases were not significantly different from the total flavonoids using chemical extraction. The contents of total flavonoids in Changzhi II were higher than that of Aijiao Bawangbian both before and after the digestion. Thus, both phenolics and flavonoids varied with periodical digestion. Phenolics and flavonoids are released mainly in small and large intestines.

**Table 1:** Phenolic and flavonoid contents of chemical extracts and *in vitro* digestion of sesame seeds.

Varieties			Phenolics (mg GAE/100 g, DW)	Flavonoids (mg CE/100 g, DW)
Changzhi II	Chemical extraction	Free	168.94 ± 3.79^h^	941.43 ± 60.21^bcd^
		Bound	77.24 ± 1.57^m^	213.05 ± 37.86^g^
		Total	246.18 ± 4.63^g^	1154.48 ± 28.84^b^
	Digestion	Oral	113.54 ± 2.02^j^	162.52 ± 58.06^g^
		Gastric phase	512.05 ± 0.74^d^	750.30 ± 139.48^def^
		Small intestine	790.34 ± 4.97^a^	1827.17 ± 403.39^a^
		Large intestine	750.91 ± 7.66^b^	1076.36 ± 298.95^bc^
Aijiao Bawangbian	Chemical extraction	Free	94.89 ± 1.64^l^	539.75 ± 64.16^f^
		Bound	43.69 ± 2.27^n^	74.28 ± 2.82^g^
		Total	138.58 ± 0.85^i^	614.04 ± 64.56^ef^
	Digestion	Oral	105.14 ± 0.94^k^	74.40 ± 8.50^g^
		Gastric phase	343.41 ± 1.80^f^	660.07 ± 13.66^ef^
		Small intestine	530.46 ± 4.78^c^	540.12 ± 86.29^f^
		Large intestine	504.71 ± 1.19^e^	834.15 ± 51.37^cde^

Values within a column that do not share a common superscript letter differ significantly (*P* < 0.05).

Fawole et al. (2015) reported a significant (*P* < 0.05) increase of total phenolic content from pomegranate material at gastric phase digestion. Ti et al. (2015) also indicated that after *in vitro* digestion, total phenolic and flavonoid contents of rice increased by 195.6 % and 34.6 %, respectively. Bouayed et al. (2011) demonstrated that 65 % of phenolics and flavonoids of apples were released during gastric digestion and were further released (<10 %) during intestinal digestion. The above findings agree with the results of our study, indicating that gastrointestinal digestion released the phenolics and flavonoids, which reached its highest level after small intestine digestion. The release of the bioactive after digestion could be due to the combination of enzymatic hydrolysis (*e.g.*, by pepsin, pancreatin) and pH changes during digestion, which can disrupt cell wall structures, cleave ester linkages, or liberate glycosylated phenolics from the food matrix, thereby increasing their extractability (Chen et al., 2020). Additionally, many phenolic acids have ionizable functional groups. Under more alkaline pH (as in the small intestine), deprotonation can increase their solubility in aqueous media (Subbiah et al., 2024). This helps liberate phenolics that were previously less soluble or trapped in the food matrix.

### 3.2 Individual phenolics

The result of six phenolic compounds including sesamol, pinoresinol diglucoside, ferulic acid, pinoresinol, sesamin and sesamolin detectable in sesame seeds are presented in Table 2. The content of free phased phenolic compounds was higher than that of the bound phenolic compounds. During the digestion phases, the ranges of the contents of sesamol, pinoresinol diglucoside, ferulic acid, pinoresinol, sesamin and sesamolin in Changzhi II were ND-2.97, ND-12.81, 2.75–9.00, 26.61–108.99, ND-77.62, ND-17.10 mg/100 g DW, respectively. During the digestion phases, the ranges of the contents of sesamol, pinoresinol diglucoside, ferulic acid, pinoresinol, sesamin and sesamolin in Aijiao Bawangbian were ND-3.69, ND-23.71, 2.76–12.51, 23.97–56.96, ND-80.83, ND-14.47 mg/100 g DW, respectively. The releases of sesamol and pinoresinol diglucoside during oral and gastric phases were significantly lower than that extracted by the chemical extracts. Sesamol and pinoresinol diglucoside were not found in small and large intestine phases. After large intestine digestion, the content of ferulic acid was higher than that of the chemical extract. After small intestines, the release of digestion, pinoresinol, sesamin and sesamolin were significantly higher than that extracted by the chemical solvent.

**Table 2:** The contents of individual phenolic compounds of chemical extracts and *in vitro* digestion of sesame seeds (mg/100 g DW).

Varieties			Sesamol	Pinoresinol diglucoside	Ferulic acid	Pinoresinol	Sesamin	Sesamolin	Sum
Changzhi II	Chemical extraction	Free	3.39 ± 0.16^b^	23.60 ± 0.78^d^	3.13 ± 0.02^f^	79.83 ± 1.11^b^	30.73 ± 0.32^d^	7.68 ± 0.17^d^	148.35 ± 1.63^c^
		Bound	ND	4.60 ± 0.02^g^	ND	ND	ND	ND	ND
		Total	3.39 ± 0.16^b^	28.20 ± 0.80^c^	3.13 ± 0.02^f^	79.83 ± 1.11^b^	30.73 ± 0.32^d^	7.68 ± 0.17^d^	152.95 ± 1.64^c^
	Digestion	Oral	2.07 ± 0.08^d^	12.81 ± 0.85^f^	3.67 ± 0.09^d^	26.61 ± 0.34^ef^	1.84 ± 0.10^fg^	ND	47.00 ± 0.30^i^
		Gastric phase	2.97 ± 0.09^c^	10.65 ± 2.56^f^	2.75 ± 0.02^g^	18.46 ± 0.96^g^	13.12 ± 0.35^e^	4.44 ± 0.17^e^	52.39 ± 2.89^h^
		Small intestine	ND	ND	2.75 ± 0.00^g^	103.28 ± 1.65^a^	77.62 ± 4.15^b^	17.10 ± 0.07^a^	200.75 ± 5.49^a^
		Large intestine	ND	ND	9.00 ± 0.45^b^	108.99 ± 3.91^a^	ND	ND	117.99 ± 3.97^e^
Aijiao Bawangbian	Chemical extraction	Free	4.41 ± 0.84^a^	53.98 ± 1.91^b^	3.41 ± 0.09^e^	18.59 ± 0.01^g^	70.60 ± 1.83^c^	8.87 ± 0.65^c^	159.86 ± 0.61^b^
		Bound	ND	4.59 ± 0.07^g^	ND	ND	ND	ND	ND
		Total	4.41 ± 0.84^a^	58.57 ± 1.84^a^	3.41 ± 0.09^e^	18.59 ± 0.01^g^	70.60 ± 1.83^c^	8.87 ± 0.65^c^	164.46 ± 0.59^b^
	Digestion	Oral	1.37 ± 0.21^e^	23.71 ± 2.78^d^	3.91 ± 0.12^c^	29.78 ± 4.31^e^	ND	ND	58.77 ± 5.17^g^
		Gastric phase	3.69 ± 0.05^b^	18.28 ± 1.63^e^	2.93 ± 0.04^fg^	23.97 ± 1.36^f^	3.44 ± 0.29^f^	ND	52.31 ± 1.24^h^
		Small intestine	ND	ND	2.76 ± 0.03^g^	39.68 ± 3.48^d^	80.83 ± 3.67^a^	14.47 ± 0.81^b^	137.74 ± 6.05^d^
		Large intestine	ND	ND	12.51 ± 0.09^a^	56.96 ± 2.23^c^	ND	ND	69.47 ± 2.15^f^

Values within a column that do not share a common superscript letter differ significantly (P < 0.05).

ND means “not detected”.

After the gastric digestion, sesamol content significantly decrease for being unstable to the low pH (pH < 5). The decrease of pinoresinol diglucoside content after gastric digestion might be due to the acidic hydrolysis of phenolic glycosides to aglycons after digestion (Bouayed et al., 2011). Thus, the release of pinoresinol significantly increased after *in vitro* digestion. Ferulic acid was stable to the low pH (Friedman et al., 2000), and its content gradually increased during the digestion. Sesamin was more stable than sesamolin, which could transform to sesamol and sesaminol during oil production process (Wan et al., 2014).

The significant increase in sesamolin and pinoresinol after small intestine digestion might be due to enhanced liberation from bound or conjugated forms, combined with improved solubility and micellar solubilization in the intestinal phase. In defatted sesame meal, Chen et al. (2019) found that pinoresinol increased significantly in the intestinal phase, consistent with enzymatic and acid-mediated release from the food matrix (*i.e.* cleavage of glycosidic and matrix bonds). The transition from acidic gastric pH to near-neutral intestinal pH can facilitate deprotonation of phenolic hydroxyls, improving extractability, and the presence of bile salts in the intestinal phase may solubilize sesamolin and other lignans *via* micellar systems. Although some degradation and transformation may occur, in these cases the rate of release and solubilization likely exceeds the rate of loss, resulting in a net increase in measurable lignans. Mechanistically, precursor or conjugated lignan forms (*e.g.* glycosides or masked derivatives) may also be enzymatically converted into the free lignans during digestion, further contributing to increases (Li et al., 2024).

### 3.3 ORAC values

**Fig. 1:**
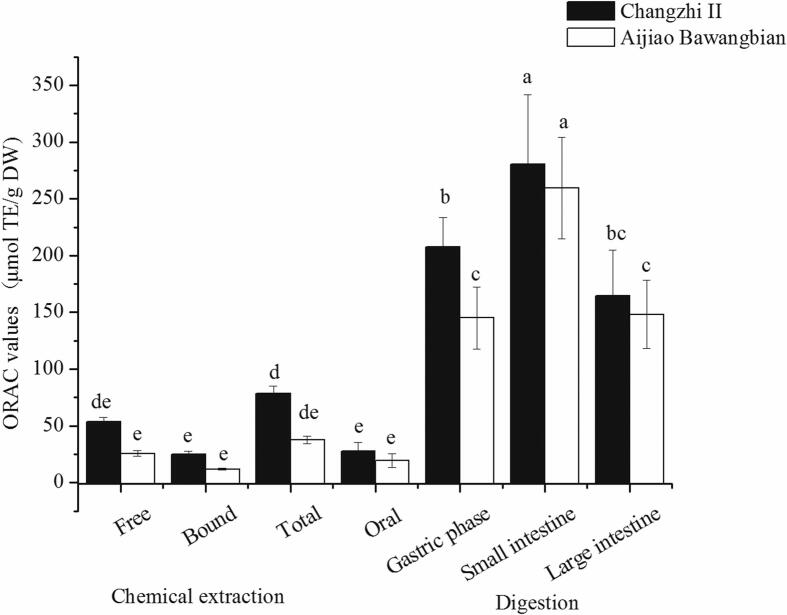
ORAC values of chemical extracts and *in vitro* digestion of sesame seeds (mean ± SD, *n* = 3). Bars with no letters in common are significantly different (*P* < 0.05).

Total ORAC values of sesame seeds before and after digestion are reported on Fig. 1. Total ORAC value of Changzhi II before digestion was 78.44 ± 6.45 μmol TE/g DW. The percentages of ORAC values of the free and bound extract contributed to the total value were 68.09 % and 31.91 %, respectively. During digestion, the ORAC value of Changzhi II varied from 27.94 (oral phase) to 280.23 (small intestine phase) μmol TE/g DW. The ORAC values of Changzhi II after gastric, small and large intestine digestion significantly increased by 165.05 %, 257.27 % and 109.59 %, respectively. Total ORAC value of Aijiao Bawangbian before digestion was 37.63 ± 3.41 μmol TE/g DW and was lower than that of Changzhi II. The percentage (68.11 %) of ORAC value of free extract contributed to the total was larger than that of bound extract (31.89 %). During digestion, the ORAC values of Aijiao Bawangbian ranged from 19.57 (oral phase) to 259.62 (small intestine phase) μmol TE/g DW, which were lower than that of Changzhi II after corresponding digestion phases. The ORAC values of Aijiao Bawangbian after gastric, small and large intestine digestion significantly increased by 285.46 %, 589.98 % and 293.87 %, respectively.

Ti et al. (2015) reported that after digestion, the total ORAC values increased by 185.7 % and 293.4 % in brown and polished rice, respectively. Chen et al. (2014) evaluated the antioxidant capacities of 33 fruits before and after the digestion, which revealed that apple, pear and cantaloupe increased during *in vitro* digestion. The study of Chiang et al. (2013) indicated that digested gooseberry showed higher antioxidant activities. The antioxidant capacities of water and 70 % methanol extracts increased after gastrointestinal digestion (Silva et al., 2013). The above research was consistent with our study. The increase of ORAC values might contribute to the enhancement of phenolics and flavonoids.

### 3.4 CAA values

**Fig. 2:**
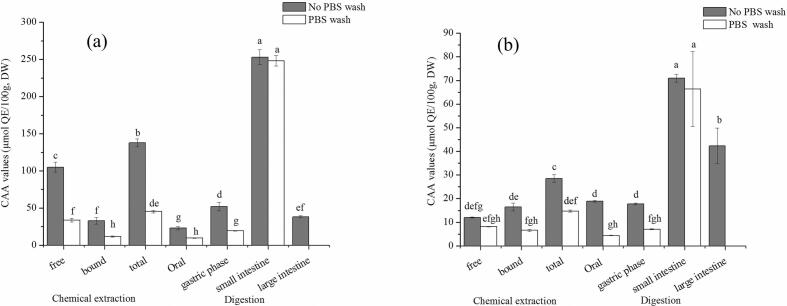
CAA values of chemical extracts and *in vitro* digestion of sesame seeds (mean ± SD, n = 3). Bars with no letters in common are significantly different (*P* < 0.05). (a) Changzhi II; (b)Aijiao Bawangbian.

A significant change in the CAA values of sesame seeds *in vitro* digestion was observed (Fig. 2). The total CAA values of Changzhi II were 137.79 ± 5.04 and 45.36 ± 1.80 μmol QE/100 g DW in no PBS and PBS wash protocols, respectively. After *in vitro* digestion, the CAA values of Changzhi II varied from 22.89 (oral phase) to 253.12 (small intestine phase) μmol QE/100 g DW and from 9.64 (oral phase) to 248.32 (small intestine phase) μmol QE/100 g DW for no PBS and PBS wash protocols, respectively. Total CAA values of Aijiao Bawangbian were 28.55 and 14.73 μmol QE/100 g DW of no PBS and PBS wash. After *in vitro* digestion, the CAA values of Aijiao Bawangbian ranged from 17.74 (gastric phase) to 70.97 (small intestine phase) μmol QE/100 g DW and from 4.40 (oral phase) to 66.40 (small intestine phase) μmol QE/100 g DW in no PBS and PBS wash protocols. After small intestine digestion, CAA values of Changzhi II increased by 83.70 % and 447.47 % in no PBS and PBS wash. After small intestine digestion, CAA values of Aijiao Bawangbian increased by 148.58 % and 350.86 % in no PBS and PBS wash, respectively. The CAA values of Changzhi II and Aijiao Bawangbian after small intestine digestion were higher than that of chemical extracts. Compounds was closely associated with cell membranes or ingested by the cells to have antioxidant activities when conducting a PBS wash (Wolfe & Liu, 2007). Interestingly, after small intestine digestion, no difference was found in the CAA values between no PBS and PBS wash, suggesting that bioaccessible substances from sesame seeds may have interacted with or been internalized by HepG2 cells, however, this remains a hypothesis requiring further validation.

Faller et al. (2012) reported that CAA value in digested *feijoada* was higher than undigested. Chinese bayberry underwent digestion present higher CAA values than extracts (Huang et al., 2014). The cellular antioxidant activity of certain foods increased after digestion. The difference could be traceable in the change of the quantities and species of compound after digestion.

### 3.5 Antiproliferative activities

Table 3 presents the antiproliferative activities of sesame seed undergoing gastrointestinal digestion on HepG2 cancer cells. The EC_50_ significantly decreased after the oral and small intestine digestion, which indicated that the antiproliferative activities of sesame seeds strengthen after oral and small intestine digestion. However, after the gastric and large intestine digestion, sesame seeds showed no significant antiproliferative activity. After digestion, the total phenolic and flavonoids content in small intestine phase both increased greatly, this might contribute to the improved bioactivity. In Fig. S1, two main peaks were found to significantly increase in small intestine, which could contribute to the increase of the total phenolic and flavonoids content and thus increase the bioactivity. Furthermore, the antiproliferative activity of Changzhi II was stronger than that of Aijiao Bawangbian, which could be due to the higher content of the total phenolic and flavonoids content in Changzhi II.

**Table 3:** The EC_50_ of antiproliferative activities of the chemical extracts and *in vitro* digestion of sesame seeds (mg/mL).

	Free	Bound	Oral	Stomach	Small intestine	Large Intestine
Changzhi II	67.86 ± 4.41^d^	142.67 ± 2.54^c^	57.15 ± 2.97^d^	ND	0.89 ± 0.23^f^	ND
Aijiao Bawangbian	181.33 ± 16.90^b^	242.43 ± 10.95^a^	37.32 ± 4.28^e^	ND	0.48 ± 0.10^f^	ND

Values within a column that do not share a common superscript letter differ significantly (P < 0.05).

ND means “not detected”.

Zhang and Liu (2015) studied the antiproliferative activity of foxtail millet on HepG2. The EC_50_ in bound extract of Jingu 34 and Jingu 28 were 40.62 and 72.03 mg/mL, respectively. The EC_50_ of the free extract of Jingu 34 and Jingu 28 were 84.53 and 147.64 mg/mL, respectively. Zhu et al. (2015) indicated that the EC_50_ for HepG2 liver cancer cells of highland barley were 67.17–99.93 and 66.40–159.5 mg/mL in free and bound extracts, respectively. Compared to the previous studies, the antiproliferative activities of sesame seeds before *in vitro* digestion were weaker than that of foxtail millet and highland barley extracts. However, the antiproliferative activities of sesame seeds after small intestine digestion were stronger than that of the foxtail millet and highland barley extracts. The difference might be attributed to the fact that the phenolic content of sesame seed after small intestine digestion was higher than that of the foxtail millet and highland barley extracts.

In our work, the concurrent peak of CAA and antiproliferative effect in the small intestinal samples aligns with evidence from recent studies showing that intestinal digestion can enhance both antioxidant capacity and growth-inhibitory effects. For example, Li et al. (2020) demonstrated that the digestion promoted the CAA value and antiproliferative activity of red-fleshed apple peels and flesh relative to the extracts. High CAA likely reflects both the release or survival of phenolic antioxidants in the small intestine and the generation or presence of metabolites capable of modulating proliferation-related pathways (*e.g.*, by influencing ROS levels, cell cycle regulatory genes).

### 3.6 Bioactive activities of six compounds of sesame seed

Table 4 shows the bioactivities of six individual phenolic compounds in sesame seeds. The decreasing order of ORAC values were ferulic acid, sesamol, pinoresinol, pinoresinol diglycoside, sesamolin, and sesamin. Only ferulic acid and sesamol showed cellular antioxidant activity. The CAA value of ferulic acid was higher than that of sesamol. Sesamol, pinoresinol and ferulic acid presented significantly antiproliferative activities with EC_50_ of 1.13 ± 0.16, 1.85 ± 0.05 and 5.14 ± 0.05, respectively. The results indicated that the antioxidant and antiproliferative activities of sesamol and ferulic acid were better than the other four individual compounds. After small intestine digestion, ferulic acid, pinoresinol, sesamolin, and sesamin remained bioaccessible. Among these, ferulic acid, owing to its higher stability and stronger bioactivity, likely contributed to the antioxidant and antiproliferative activities observed in the small intestine samples. Ferulic acid, a simple cinnamic-type phenolic, has demonstrated good stability and high measured bioaccessibility in multiple simulated gastric and intestinal digestion studies (Sánchez-Velázquez et al., 2021). Moreover, the intestinal environment (near-neutral pH, presence of bile salts and pancreatic enzymes) promotes micellar solubilization and release from the food matrix, increasing extractability of these relatively small phenolics compared with larger or more labile lignans. Taken together, the combination of favorable solubility, pH stability in the intestinal range, and enhanced micellar solubilization likely explains why ferulic acid retain measurable activity after intestinal digestion.

**Table 4:** ORAC values, CAA values, antiproliferative activities, and cytotoxicity of selected six compounds of sesame seed.

	ORAC (μmol TE/mM)	No PBS wash (μmol QE/mM)		PBS wash (μmol QE/mM)	EC_50_ (mM/L)	CC_50_ (mM/L)
Sesamol	560.59 ± 5.84^b^	45.07 ± 2.01^b^		1.79 ± 0.12^b^	1.13 ± 0.16^c^	>6
Pinoresinol Diglycoside	158.43 ± 18.65^d^	ND		ND	>4	>4
Ferulic acid	2367.15 ± 90.15^a^	77.40 ± 10.67^a^		53.78 ± 12.74^a^	5.14 ± 0.05^a^	>6
Pinoresinol	278.26 ± 6.92^c^	ND		ND	1.85 ± 0.05^b^	>2
Sesamin	29.59 ± 12.97^e^	ND		ND	>2.5	>2.5
Sesamolin	107.12 ± 10.17^d^	ND		ND	>4	>4

Values within a column that do not share a common superscript letter differ significantly (P < 0.05).

ND means “not detected”.

Previous studies have stated that sesamol, sesamin, sesamolin, ferulic acid, and pinoresinol showed different antioxidant activities when examined by the DPPH free radical scavenging assay (Leu et al., 2011; Ou & Kwok, 2004; Suja et al., 2004). Wolfe and Liu (2007) had reported that ferulic acid exhibited cellular antioxidant activity. Deng et al. (2013) found that sesamin could reduce HepG2 cell growth through regulating G2/M phase arrest and apoptosis. The studies above proved that the compounds exited in sesame seed could be the good resource of natural antioxidant.

Correlations between the total content of the six compounds and the CAA values of the no PBS (*r* = 0.698, *P* < 0.05) and PBS protocol (r = 0. 684, *P* < 0.05) of sesame seeds were significant, which indicated that the six compounds of sesame seed significantly contributed to the cellular antioxidant capacities. There were no significant correlations between the total content of the six compounds and the antiproliferative activities of sesame seeds. The six compounds investigated in the study might weakly contribute to the total antiproliferative activities of sesame seeds. Other compounds including anthraquinone (Furumoto & Nishimoto, 2016), phytosterols (Bradford & Awad, 2007), and black sesame pigment (Panzella et al., 2012) in sesame seeds have been reported to be anti-cancerous, which need further research.

## 4 Conclusion

The current study investigated the impact of the *in vitro* gastrointestinal digestion of sesame seed on phenolics, flavonoids, ORAC value, CAA value and antiproliferative activity in HepG2 cells. The phenolics, flavonoids and ORAC value of sesame seed increased gradually during *in vitro* digestion. Sesame seed had considerable CAA and antiproliferative activity both before and after small intestine digestion. In tested phenolics, sesamol and ferulic acid showed better antioxidant and antiproliferative activities than other four compounds in cellular level. In this study, black sesame exhibited stronger activity than white sesame. However, these findings may be influenced by multiple factors including seed variety, origin, and storage history, which should be considered in future investigations.

## CRediT authorship contribution statement

**Lin Zhou:** Writing – original draft, Investigation, Funding acquisition, Conceptualization. **Xiaohui Lin:** Writing – review & editing, Investigation, Funding acquisition, Formal analysis, Conceptualization. **Ruixue Guo:** Investigation, Formal analysis. **Tong Li:** Formal analysis. **Charles Brennan:** Writing – review & editing. **Xiong Fu:** Resources, Funding acquisition. **Rui Hai Liu:** Writing – review & editing, Supervision, Resources, Funding acquisition.

## Declaration of competing interest

The authors declare that they have no known competing financial interests or personal relationships that could have appeared to influence the work reported in this paper.

## Data Availability

Data will be made available on request.

## Appendix A Supplementary data

Supplementary material: Original HPLC profiles of sesame seeds Image 1
