# Supplementary material for: Phytochemical compounds, antioxidant activity, and antiproliferative activity of sesame seeds as affected by simulated digestion
Source: Food Chem X. 2025 Nov 26;32:103317. doi: 10.1016/j.fochx.2025.103317 (PMC12719202; doi:10.1016/j.fochx.2025.103317)
Supplement: Supplementary file 1 — Supplementary material: Original HPLC profiles of sesame seeds [file mmc1.docx]

**Phytochemical compounds, antioxidant activity, and antiproliferative activity of sesame seeds as affected by simulated digestion**

Lin Zhou^a,b^, Xiaohui Lin^*c,d^, Ruixue Guo^a,b^, Tong Li^e^, Charles Brennan^f^, Xiong Fu^b^, and Rui Hai Liu^*e^

^a^School of Life Science and Biopharmaceutics, Guangdong Pharmaceutical University, Guangzhou, 510006, People’s Republic of China.

^b^School of Food Science and Engineering, South China University of Technology, Guangzhou, 510640, People’s Republic of China.

^c^School of Biosystems and Food Engineering, University College Dublin (UCD), Belfield, Dublin 4, Ireland

^d^Ghent University, Center for Microbial Ecology and Technology, B-9000, Gent, Belgium

^e^Department of Food Science, Cornell University, Ithaca, NY 14850-2824, USA.

^f^School of Science, RMIT University, Melbourne VIC 3000, Australia

*****Corresponding author

Xiaohui Lin

E-mail: xiaohui.lin@ucd.ie

Rui Hai Liu

E-mails: rl23@cornell.edu (R.H.L.)

Tel.: +1-607-255-6235; Fax: +1-607-255-4868

Black sesame free compound extraction

Black sesame oral phase

Black sesame gastric phase

Black sesame small intestine

Black sesame large intestine

White sesame free compound extraction

White sesame oral phase

White sesame gastric phase

White sesame small intestine

White sesame large intestine

Fig. S1. HPLC original profiles of sesame seeds
